# Prevalence and associated factors of post-traumatic stress disorder (PTSD) among a cohort of Srilankan post-partum mothers: a cross-sectional study

**DOI:** 10.1186/s12884-021-04058-z

**Published:** 2021-09-17

**Authors:** Wedisha Imal Gankanda, Ileperuma Arachchige Gayani Malsha Perera Gunathilake, Nalaka Lasantha Kahawala, Augustus Keshala Probhodana Ranaweera

**Affiliations:** 1 De Soysa Hospital for Women, Colombo, Sri Lanka; 2 National Institute of Mental Health, Angoda, Sri Lanka; 3 Medical Officer of Health, Horana, Sri Lanka; 4 Department of Gynaecology and Obstetrics, Faculty of Medicine, University of Colombo, Colombo, Sri Lanka

**Keywords:** PTSD, Post-partum PTSD, Post-partum depression, PSS-SR, PDSS

## Abstract

**Background:**

Post-Traumatic Stress Disorder (PTSD) usually follows a catastrophic event. However, the experience of child birth can be severe enough to cause PTSD in some women. The aim of this study is to highlight the prevalence of Post Traumatic Stress Disorder among a cohort of postpartum mothers.

**Methods:**

A cross-sectional study was conducted in field clinics of a semi-urban area in Sri Lanka. A pre-tested interviewer administered checklist was used to collect socio-demographic and pregnancy related data. Pre-existing self-administered, validated Sinhalese versions of the Edinburgh Postnatal Depression Scale (EPDS) and PTSD Symptom Scale-Self Report (PSS-SR) were used to assess the presence of Post-Partum Depression (PPD) and PTSD, respectively. Each participant was assessed for PTSD and PPD after one, two and six months following delivery. Scores of PPD > 9 and PSS-SR > 13 were taken as screening positive for the two conditions, respectively.

**Results:**

Two hundred and twenty-five mothers at the end of postpartum one month were recruited for the study. The response rate at their follow-up visits at the second and sixth months were 95 % (*n* = 214) and 93 % (*n* = 211). The prevalence of postpartum PTSD was 2.7 % (n = 6), 0.9 % (n = 2) and 0.5 % (n = 1) after one, two and sixth months respectively. Prevalence of postpartum PTSD was 3.6 % over 6 months. Verbal abuse during labour (*p* = 0.04) and the presence of postpartum depression (*P* ≤ 0.001) were significantly associated with postpartum PTSD. There were no significant associations between PTSD and gestational age at delivery, index pregnancy being a planned pregnancy, a history of subfertility, family history of psychiatric disorders, intimate partner violence, receiving antenatal counseling, type and mode of delivery, duration of labour, presence of a labour companion, post-partum hemorrhage, manual removal of placenta, negative birth experience, low APGAR score of the baby at delivery, receiving neonatal and maternal intensive care, birth defects, problems with breast feeding or opportunity to discuss with a health care worker.

**Conclusions:**

Prevalence of postpartum PTSD in this community-based study is 3.6 %; which is comparable with the overall global prevalence. PTSD was significantly associated with verbal abuse during labour and postpartum depression.

## Background

“Stress” is defined as a response to a change in the environment, which may be adaptive or non-adaptive. A non-adaptive stress response like Post Traumatic Stress Disorder (PTSD) can impair normal functionality [1].

PTSD is characterized by a symptomatic triad of re-experiencing (flashbacks, nightmares), avoidance (staying away from reminders) [2] and arousal (reactive sweating, palpitations). PTSD typically presents a few weeks to several months after exposure to an exceptionally shocking, threatening or catastrophic event, in the absence of an organic cause.

Traumatic child birth is a recognized risk factor for developing postpartum PTSD [3]. Birth is a life-changing event that can be daunting with intense physical and mental stressors and feelings of mental trauma, influencing the potential development of postpartum PTSD. Furthermore, postpartum PTSD is a condition associated with Post-Partum Depression (PPD)[3], negative relationships between couples [1, 4], fertility issues [5] violence and suicide [3] in the mother. In neonates, effects such as low birth weight, lower rates of breastfeeding, negative effects on development and mother-infant relationship can occur[6]. It is imperative to identify the mothers who develop postpartum PTSD, as prompt intervention can benefit the mother, partner and the newborn.

Universal screening in postpartum mothers for postpartum depression (PPD) is currently practiced in this study setting. However, there is no such system to screen for post-partum PTSD. Since most scales used for PTSD are not specific to birth, there is an urgent need to develop specific tools to facilitate the identification of this condition.

Postpartum PTSD had been a subject of extensive study worldwide with a global prevalence of 3.17 % [7]. However, published data is very limited within the South Asian region and absent in Sri Lanka. Therefore, it is important to determine the hidden burden of PTSD and the factors associated with it in order to take necessary remedial actions.

This study was conducted with a view of describing the prevalence and associated factors of Post Traumatic Stress Disorder among a cohort of postpartum mothers.

## Methods

A cross sectional study at three different points of time, with an analytical component, was conducted at four randomly selected field clinics located in the Horana MOH area. Four clinics were selected by simple random sampling out of nine polyclinics.

Minimum sample size was calculated using the formula to calculate the sample size for prevalence / cross sectional studies considering power, type 1 and type 2 errors. The minimum sample size calculated was 180 using the prevalence of 7.8 % (in a study done in Israel)[8]. By considering non respondent rate and feasibility, 225 was taken as the final sample size[9].

Two hundred and twenty-five postpartum mothers, without a prior history of psychiatric illnesses, were recruited for the study at one month after delivery.

Data collection was done using an interviewer administered checklist (for socio-demographic data) and two Sinhalese validated self-administered questionnaires. Firstly, the PTSD Symptom Scale-Self Report (PSS-SR) [10] was used to assess PTSD symptoms and secondly the Sinhalese validated Edinburgh Postpartum Depression Screening Scale: (EPDS) [11] was used to assess for postpartum depression. Both these questionnaires have been used in similar studies in Sri Lanka [10, 11]. Follow up at the end of second and sixth months were done using the same self-administered questionnaires. It’s debatable whether PTSD should be seen as a dichotomy or a continuum. Even though the cases identified and percentages of women meeting specific criteria differ according to the instrument used, many women clearly suffer from post-traumatic stress symptoms following child-birth. For the purpose of the study the dichotomous cut off of PSS-SR score > 13 was adapted[12]. Its noteworthy that PSS-SR is a symptom measure, not a diagnostic tool.

A pilot study was conducted in a polyclinic in the Horana MOH area, which was not included in the study in order to assess the practicality and feasibility in carrying out the data collection. A specially trained medical officer coordinated data collection at all times, ensuring uniformity of given information and sensitive communication. Affected mothers were appropriately escalated to specialist perinatal mental health team ensuring patient safety.

Ethical clearance was obtained from the Ethical Review Committee of the Faculty of Medicine, University of Colombo (Ref No:EC-16-169). The information sheet was explained to each participant and verbal consent was obtained.

Verbal consent was deemed more appropriate by the Ethical Review Committee, since a state-led universal post-partum screening (using EPDS) program was already in place at the same clinics, for which verbal consent alone was being taken. This similar non-interventional study has no more than minimal risks to its subjects. Considering the study context and setting, verbal consent was considered sufficient and appropriate.

Significance of the associations was tested with the proportions. Fishers exact test was used, since the Chi square was not appropriate due to less five observations in most of the cases. Statistical analysis was performed using the SPSS statistical package version 18.

## Results

Two hundred and twenty-five mothers at one-month post-partum were included in the study. Out of them 214 (95.1 %) and 211(93.8 %) were re–evaluated at the second- and sixth-months post-partum with minimal lost to follow up. The mean age of the mothers was 28.38 years (SD = 5.52), while the median age was 28 years (range 15–42 years). Among the participants, 40.9 % (n = 92) had an education level above the GCE ordinary level (O/L).

The prevalence of PPD was 7.1 % (n = 16), 4.2 % (n = 9) and 0.9 % (n = 2) at the first, second and sixth month postpartum, respectively. Altogether 24 (10.4 %) had developed PTSD following delivery during first 6 months.

### Prevalence of postpartum PTSD

**Fig. 1:**
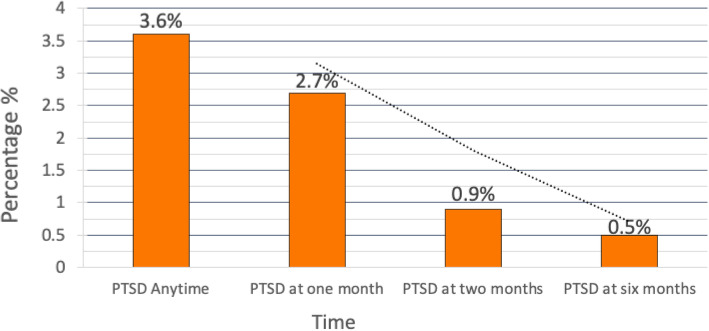
Prevalence of PTSD during study period

Figure 1 summarizes the prevalence of PTSD during the study period.

At the first, second and sixth month postpartum, the prevalence of PTSD was 2.7 % (n = 6), 0.9 % (n = 2) and 0.5 % (n = 1), respectively. Altogether 8 (3.6 %) had developed PTSD following delivery up to 6 months.

### Factors associated with postpartum PTSD

#### Pre-pregnancy factors

Table 1 depicts the association between postpartum PTSD and pre-pregnancy factors; family history of mental illness, level of education of the mother and intimate partner violence were not significantly associated with postpartum PTSD.

**Table 1:** Pre-pregnancy factors and association with postpartum PTSD

Factor		PTSD		Total	Fishers’ exact test (2-sided) ***p*** value *
		Yes	No		
		No. (%)	No. (%)	No.	
Family history of mental illness	Yes	0 (0)	5(100)	5	*p*=1.00
	No	8(3.6)	212(96.4)	220	
Level of education	<=O/L	6(4.5)	126(95.5)	131	*p*= 0.48
	>O/L	2(2.1)	91(97.9)	92	
Intimate Partner violence	Yes	0(100)	3(100)	3	*p*=1.00
	No	8(3.6)	214(96.4)	222	
**Total**		**8**	**217**	**225**	

*Fishers test used since there are some cells with values < 5, *p*=*p* value

#### Antenatal factors

Table 2 describes the antenatal factors associated with postpartum PTSD. None of the factors included were significantly associated with the development of postpartum PTSD.

**Table 2:** Antenatal factors and association with postpartum PTSD

Factor		PTSD			Total	Fishers’ exact test (2-sided) *p* value*
		**Yes**		**No**		
		**No. (%)**		**No. (%)**	**No.**	
Parity	1	6 (6.3)	90(93.8)		96	*p* = 0.08
	> 1	2(1.6)	127(98.4)		129	
Planned pregnancy	Yes	4(3)	128(97)		132	*p* = 0.72
	No	4(4.3)	89(95.7)		93	
History of subfertility	Yes	0(0)	17(100)		17	*p* = 1.00
	No	8(3.8)	200(96.2)		208	
Number of antenatal clinic visits	< 4	1(6.7)	14(93.3)		15	*p* = 0.47
	>=4	7(3.4)	201(96.6)		208	
Receiving antenatal advice	Yes	8(3.7)	209(96.3)		217	*p* = 1.00
	No	0	8(100)		8	
Inter-pregnancy interval(years)^a^	<=2	0(0)	12(100)		12	*p* = 1.00
	> 2	2(1.7)	115(98.3)		117	
**Total**		**8**	**217**			

*Fishers test used since there are some cells with values < 5, *p* = *p* value, ^a^Total = 129 since Interpregnancy interval is not applicable for primigravidae

#### Intra-partum factors

Table 3 describes the association of intra-partum factors with postpartum PTSD. Verbal abuse in labour was significantly associated with postpartum PTSD.

**Table 3:** Association of intra-partum factors with postpartum PTSD

Factor		PTSD			Total	Fishers’ exact test (2-sided) *p* value*
		**Yes**		**No**	**No.**	
		**No. (%)**		**No. (%)**		
Maturity at birth	Preterm	2 (1.6)	127(98.4)		129	*p* = 0.06
	Term	6(6.3)	90(93.8 %)		96	
Type of hospital	State	7(3.7)	184(96.3)		191	*p* = 1.00
	Private/other	1(2.9)	33(97.1)		34	
Fear of anticipated pain	Yes	4(8)	46(92)		50	*p* = 0.054
	No	4(2.3)	171(97.7)		175	
Verbal abuse during labour	Yes	1(12.5)	7(87.5)		8	*p* = 0.04
	No	2(0.9)	215(99.1)		217	
Physical abuse during labour	Yes	0(0)	1(100)		1	*p* = 1.00
	No	8(3.6)	216(96.4)		224	
Presence of labour companion	Yes	1(2.6)	37(97.4)		38	*p* = 1.00
	No	7(3.7)	180(96.3)		187	
Mode of delivery	Vaginal	3(2.3)	125(97.7)		128	*p* = 0.50
	Vacuum/Forceps	0(0)	5(100)		5	
	EL-LSCS	4(4.9)	78(95.1)		82	
	EM-LSCS	1(10)	9(90)		10	
Perception of labour as negative experience	Yes	0(0)	10(100)		10	*p* = 1.00
	No	8(3.6)	207(96.3)		215	
Duration of active labour(hours)^a^	< 4	3(2.5)	119(97.5)		122	*p* = 0.29
	4–8	1(3.8)	25(96.2)		26	
	> 8	1(12.5)	7(87.5)		8	
**Total**		**8**	**217**		**225**	

*Fishers test used since there are some cells with values < 5, *p* = *p* value

^a^ Total = 156 since some mothers underwent CS prior to labour

#### Postpartum Factors

Table 4 describes the postpartum factors associated with postpartum PTSD. Concomitant postpartum depression was significantly associated with postpartum PTSD.

**Table 4:** Postpartum factors and association with postpartum PTSD

Factor		PTSD			Total	Fishers’ exact test (2-sided) *p* value*
		**Yes**	**No**		**No.**	
		**No. (%)**	**No. (%)**			
PPH	Yes	0(0)		8(100)	8	*p* = 1.00
	No	8(3.6)		209(96.4)	217	
Manual removal of placenta	Yes	0(0)		7(100)	7	*p* = 1.00
	No	8(3.6)		210(96.4)	218	
Baby cried after birth	Yes	8(3.7)		208(96.3)	216	*p* = 1.00
	No	0(0)		9(100)	9	
Mother: ICU admission	Yes	0 (0)		6(100)	6	*p* = 1.00
	No	8(3.6)		211(96.4)	219	
Baby: PBU admission	Yes	1(4.5)		21(95.5)	22	*p* = 0.57
	No	7(3.4)		196(96.6)	224	
Baby: NICU admission	Yes	1(4.7)		20(95.3)	21	*p* = 0.55
	No	7(3.4)		197(96.6)	204	
Baby: Presence of birth defects	Yes	0(0)		14(100)	14	*p* = 1.00
	No	8(3.7)		203(97.7)	211	
Breast feeding	Yes	8(3.6)		211(96.7)	219	*p* = 1.00
	No	0(0)		6(100)	6	
Family support	Yes	7(3.2)		212(96.8)	219	*p* = 0.20
	No	1(1.7)		5(98.3)	6	
Readmission	Yes	2(6.4)		29(93.6)	31	*p* = 0.30
	No	6(3.1)		188(96.9)	194	
Physical ailments	Yes	3(8.1)		34(91.7)	37	*p* = 0.13
	No	5(2.6)		183(97.3)	188	
Opportunity to discuss concerns	Yes	2(2.8)		69(97.2)	71	*p* = 1.00
	No	6(3.9)		148(96.1)	154	
Postpartum depression	Yes	6(25)		18(75)	24	***p* < 0.001**
	No	2(1)		199(99)	201	
**Total**		**8**		**217**	**225**	

*Fishers test used since there are some cells with values < 5, *p* = *p* value

## Discussion

In this study, the prevalence of PTSD at one, two and six months postpartum, was 2.7 % (n = 6), 0.9 % (n = 2) and 0.5 % (n = 1) respectively while the overall prevalence was 3.6 %. These figures are comparable to the prevalence of PTSD in most of the studies in other countries. In one metanalysis prevalence of postpartum PTSD in community samples was estimated to be 3.1 % and in at-risk samples 15.7 %[13]. Another meta-analysis including 50 studies (N = 21, 429) from 15 countries showed a global prevalence of 3.17 % at one month postpartum, which is compatible with the results of our cohort [7].

A study using the same scale( PSS-SR) used in this study in USA, found a higher prevalence of postpartum PTSD ( 9 %) after 1 month, however there were no further follow up data in the study [14].

A cross sectional study done at a fetal high risk unit in Brazil, using a different scale (PCL-C), found a higher prevalence (9.4 %) of post-partum PTSD at one month [15].

A Serbian study using a different scale (Clinician-Administered PTSD Scale ), shows a lower prevalence (2.4 %) of postpartum PTSD at one month and no prevalence at the end of second and the third months from delivery.[10].

A Dutch study [16] involving 428 women shows a prevalence of 1.2 % while a Swedish study( N = 1640) using TES, shows a prevalence of 1.7 % for postpartum PTSD [17].

Socio-economic and cultural factors, differences in obstetric care models and the usage of different scales may play a role in these observed differences.

In this study, only two significant associations with postpartum PTSD were demonstrated; i.e. Verbal abuse during labour (p = 0.04) and presence of Postpartum Depression (PPD) (P = < 0.001). Very few studies have specifically looked into the effect of verbal abuse during labour on the development of PTSD [4] whereas there are many studies including a meta-analysis describing PPD as a risk factor for postpartum PTSD [5–7, 12]. In this cohort, overall prevalence PPD was of 10.7 % over six months. These cases could be new onset and /or undetected as well as primary or secondary.

According to studies worldwide, several factors associated with the development of PTSD following childbirth have been identified. Among the known pre-pregnancy associated factors, low educational level [18], unplanned pregnancy [14],fear of child birth [7], family history of mental disease [7], nulliparity [17] or parity > 3 [15] were not found to be significantly associated with postpartum PTSD in this study.

Intra-pregnancy factors associated with postpartum PTSD include gestational age at delivery [18], number of antenatal care visits [18], poor health or complications in pregnancy [7], hospital admission due to pregnancy complications [19], fear of childbirth [15], expected intense pain [15] and intimate partner violence [15]. However, none of these factors showed a significant association with postpartum PTSD in this study.

Reported intra-partum factors associated with postpartum PTSD are duration of labour [18], mode of delivery [18], negative subjective birth experiences [7], having an operative birth (operative vaginal or caesarean section) [7, 19], lack of support [7] and poor maternal experience of control during childbirth [19]. However, none of these factors were significantly associated with PTSD in this study.

Among documented postpartum factors found to be associated with postpartum PTSD are poor neonatal outcome [15], low neonatal APGAR Score at delivery [7], neonatal and maternal intensive care [5], birth defects [4], lack of support from family and partner [7, 14], postpartum physical problems [14], lack of exclusive breast feeding at one month [14] and not having an opportunity to discuss concerns with health care staff about mental wellbeing [14]. In our study, none of these associations reached the level of significance. Possible reasons for the lack of association may be due to a lower prevalence of PTSD.

Being a small sample size, there are limitations in generalizing these results to the entire population of the country, where social and economic backgrounds might differ. Since this is a cross sectional study, it is difficult to establish the exact temporal relationships between the associated factors (e.g. when PPD and PTSD both present at the time of data collection, it is uncertain which developed earlier). PSS-SR scores less than the cut-off (Partial PTSD) were not analyzed since the used validated study instrument did not specifically categorizing them.

## Conclusions

The overall prevalence of postpartum PTSD in this cohort is 3.6 % over 6 months. It is of similar prevalence to large global studies. Out of the factors assessed in this study, only verbal abuse during labour (p = 0.04) and presence of PPD (p = < 0.001) were significantly associated with postpartum PTSD.

Authors recommend it would be beneficial to routinely screen post-partum mothers for PTSD and refer accordingly.

## Data Availability

Original data is available with the authors and data file can be accessed from the repository (https://www.synapse.org/#!Synapse:syn23583521) on request from the corresponding author.

## Abbreviations

EL: Elective
EM: Emergency
EPDS: Edinburgh Postnatal Depression Scale
GCE: General Certificate of Education
ICU: Intensive Care Unit
LSCS: Lower Segment Caesarean Section
MOH: Medical Officer of Health
NICU: Neonatal Intensive Care Unit
PBU: Premature Baby Unit
PCL-C: PTSD Check List-Cilvilian Version
PPD: Post-Partum Depression
PPH: Post-Partum haemorrhage
PSS-SR: PTSD Symptom Scale-Self Report
PTSD: Post-Traumatic Stress Disorder
SD: Standard Deviation
SPSS: Statistical Package for the Social Sciences
TES: Traumatic Event Scale
